# Model optimization and performance evaluation of hand cranked music box base structure manufactured via 3D printing

**DOI:** 10.1016/j.heliyon.2021.e08432

**Published:** 2021-11-20

**Authors:** Naufal Achmad Salman Alfarisi, Gil Nonato C. Santos, Rachmadi Norcahyo, Jayan Sentanuhady, Nikmatul Azizah, Muhammad Akhsin Muflikhun

**Affiliations:** a Mechanical and Industrial Engineering Department, Gadjah Mada University, Indonesia; b Physics Department, De La Salle University, Manila, Philippines; c Ghent University, Ghent, Belgium

**Keywords:** Music box, Remodeling, 3D printing, Sound quality, Angle effect

## Abstract

A sound produced from the music box could mesmerize music lovers. The complex mechanism that combined manual with semi-automatics movement creates the music box as a challenge for the manufacturer to innovate and optimize. This study focused on redesigning a hand-cranked music box's base structure using 3D printing and comparing the sound produced with the original model. It is shown that 3D printing can create a complex model with minimum material waste and good repeatability. After remodeling the music box's in a 3D CAD model, the prototype was built, and the tune played by each model was recorded and compared. The results showed that four improvements were made in the barrel mounting, crankshaft holder, crankshaft locker, and comb locker from the built four models. The sound analysis shows that the quality of sound can be improved by using the system's spacer. Furthermore, the finite element method and exact experiment results show that the loudest and best sound quality can be achieved using a 60° angle slope for the music box base structure.

## 1 Introduction

The music box is a device that was once famous for bringing automation to music at home. Depending on the local community's preferred term, it has many names like a music box, musical box, or nickelodeon [1]. Generally, a music box is a self-playing musical instrument usually actuated by winding a spring, although several models actuated by hand. Music boxes became popular around the early 1840s to mid-1890s, but some of the earliest music boxes dated to the late 1700s [1, 2]. At that time, music boxes were not the only automatic musical instrument. The story of musical automata like automatic flute players and mechanical birds can be found around the 1500–1800 era [2].

There are many music box types, from the complex one that plays multiple songs and instruments to as simple as the hand-cranked one. For example, a specific type of music box known as an orchestra box has more than just a pin-plucked comb as its musical instrument. It could contain bells, drums, and even organs that are all then hit by small hammers as the cylinder rotates. Another type of music box does not have pinned cylinder for plucking the comb. Instead, it has a disk. The form and geometry between the two types of movement mechanism, but its concept is still the same. The disk rotates the holes on it caused star wheels to strike the comb and produce the desired sounds. The disk is also interchangeable, creating the instrument to play multiple tunes depending on the disk [2]. Hand-cranked music box actuated by rotating a crankshaft by hand. The crankshaft then rotates the pinned cylinder through the barrel wheel. As it rotates, each pin will pluck the comb's teeth accordingly. The sounds produced as the teeth vibrate, the lower notes produced by longer and weighted teeth, while shorter and lighter teeth produced higher notes. The vibrations then resonate through the base body and then to the medium where the music box is put on.

Since it was first recorded about music box as a music instrument, the materials used as it structures are made from metal-based materials [3, 4]. The outer structure is the cover that can be designed and modeled as an art, often made by wood-based material [5]. This indicates that there are not so many researchers in the previous work that have a concern about the base structures of the music box due to the difficulty and sound quality of the instrument [6, 7]. On the other hand, technology has grown rapidly in the past decades and has motivated many researchers to actuate the idea into a 3D model. One of the technologies invented by researchers and are used widely at now a day is 3D printing as part of the additive manufacturing process.

Additive manufacturing (3D printing) is a method of manufacturing that features added materials layer by layer and forms the desired model [8]. This method makes manufacturing a 3D model straight from CAD files possible. The process starts by heating a filament, usually made of a composite of some kind, until it melts that then extruded through a nozzle to a heated bedplate. The bed plate is heated to a certain degree where the filament is hot enough to stick to the bedplate and cool enough to harden [9]. The filament materials used in 3D printing have various types with multifunction applications [10, 11, 12]. Several researchers used natural fiber as filament materials made of Flax, Jute, Kenaf Coir, Cotton Wood, Bamboo, and Hemp as the filament materials [10], where He et al. [13] used carbon fiber and special ink as filaments to create fiber-based composites. Furthermore, in terms of polymer-based materials, Fu et al. [14] summarized the materials used for 3D printing. Specific materials such as acrylonitrile butadiene styrene (ABS), polylactic acid (PLA), polycarbonate (PC), polyetherimide (Ultem), polyphenylsulfone (PPSF), nylon, and polyether ketone (PEKK) were used. PLA usually used in commercial 3D printing among other filament materials used for 3D printing, that available in the market due to several advantages, i.e., bio-based and biodegradable materials, lower environmental impacts, can be recycled as biomass, PLA is more robust and stiffer than ABS, PLA is one of the easiest materials to 3D print successfully [14, 15].

In terms of applications, the 3D printing method was successfully reported to be used in many applications by researchers such as in safety instruments [16], medical and pharmaceutical [17], buildings and structures [18], cement-based material for structural applications [19], battery components [20], educational models in biological sciences [21], Biomedical and bioengineering [22], and industrial related to supply chain [23]. There are already several musical instruments manufactured with 3D printing in terms of musical instruments, either the parts and components or the whole instruments, such as guitars, drums, and saxophone [8]. Music and its instrument are more related to art and how it affects human feeling. The previous research showed that sound quality could affect the people in public space [24]. The study shows that people's expectations of loudness and comfort were related to the highest sound levels rather than the environment's time-equivalent sound levels. Furthermore, higher satisfaction levels were associated with visitors' perceptions of a quieter and more friendly soundscape.

Since 3D printing can produce various parts, especially music instruments, the related evaluation performance of the material in sound vibration becomes important. The evaluation of the vibration and sound that produced by using polymer-based materials has been evaluated by Greethamma et al. [25]. The study evaluated the Vibrations from the sound transmitted via various materials such as metals and polymers. It is shown that the vibration damping of polymers is influenced by two important factors: viscoelasticity and glass transition. Furthermore, it is shown that metal materials are able to radiate sound, while polymer materials minimize vibration and sound. Polymers' damping properties are used in vibration dampers, shock absorbers, bridge bearings, and seismic absorbers.

After reviewing the previous work, as explained in the previous section, additive manufacturing using 3D printing technology was growing rapidly, with many applications are successfully manufactured using this method included in music instruments. It is demonstrated that additive manufacturing is known for creating a complex-shaped model with high accuracy and precision. In the music instrument, sound quality is one of the key factors that are used to evaluate the various models of music components. Music quality can be defined as the assessment that can be used to evaluate the audio output from specific devices. The present paper used time vs. power, and frequency vs. power to evaluate the sound quality. Among all of the studies published by researchers, the study evaluated and analyzed the sound quality in music box manufactured using 3D printing still miss. Therefore, the preference assessment of the present study attempted to analyze and evaluate remodeling and redesign music box base structures using the 3D printing method. The study also compared the sound quality when the metal-based, PLA-based, and spacer inserter was induced into the model. The results will benefit the engineers, researchers, and musicians who used 3D printing in musical instruments.

## 2 Materials and method

### 2.1 Materials

The filament material used for the 3D printing process in this study was polylactide or also known as polylactic acid (PLA). PLA is a polymer obtained from heating lactic acid under vacuum conditions discovered by Carothers in 1932 [26]. PLA's properties that are used in the present study are shown in Table 1. In this study, the filament was manufactured by eSUN® (eSUN, Shenzhen, China). The PLA type was eSun PLA+ with a diameter of 1.75 mm. This PLA was chosen mainly because of its availability and good mechanical properties that have advantages and are suitable for model prototyping.

**Table 1:** Polylactic acid mechanical properties.

Characteristics	Unit	Amount
Tensile strength	MPa	59
Elongation at break	%	7
Elastic modulus	MPa	3500
Shear modulus	MPa	287
Poisson's ratio		– 0.36
Yield strength	MPa	70
Flexural strength	MPa	106
Unnotched izod	J/m	195
Notch izod impact	J/m	26
Rockwell hardness	HR	88
Heat deflection temp	°C	55
Vicat penetration	°C	59
Ultimate tensile strength	MPa	73
Percent of elongation	%	11.3
Young's modulus	MPa	1280

In this study, two different types of spacers are used in the music box. The spacer is used to determine the vibration and sound characteristics of the Music Box manufactured using 3D printing. The spacers' element compositions (Steel and Aluminium) are obtained using an Applied Research Laboratories (ARL) - Model 3560 - Simultaneous ICP-OES, and the results are shown in Table 2.

**Table 2:** Spacer element contents.

Element	Stainless Steel	Aluminium
	(%)	(%)
C	0.050%	0.00%
Si	0.530%	0.13%
S	0.004%	0.00%
Р	0.020%	0.00%
Mn	>11.570%	0.09%
Ni	0.560%	0.00%
Cr	>34.060%	0.01%
Mo	0.010%	0.00%
Cu	>4.910%	0.10%
W	0.000%	0.00%
Ti	0.004%	0.02%
Sn	0.004%	0.00%
Al	0.030%	98.80%
Pb	0.000%	0.01%
Ca	0.130%	0.00%
Zn	0.000%	0.16%
Mg	0.000%	0.02%
Fe	47.620%	0.66%

The remodeled music box was produced using Inventor 3D CAD software. After the model was built, Ultimaker Cura v4.8 software converted the 3D STL model to G-Code. The G-Code was then transferred to the Anycubic Mega i3 S 3D Printer to make the remodeled music box prototype. The printing setting for every model is shown in Table 3.

**Table 3:** Manufacture set up using 3D printer.

Characteristics	Unit	Amount
Layer Height	mm	0,15
Infill Density	%	100
Infill Pattern		Gyroid
Printing Temperature	°C	190
Build Plate Temperature	°C	60
Print Speed	mm/s	40
Retraction		On
Support Density	%	5

### 2.2 Method

**Figure 1:**
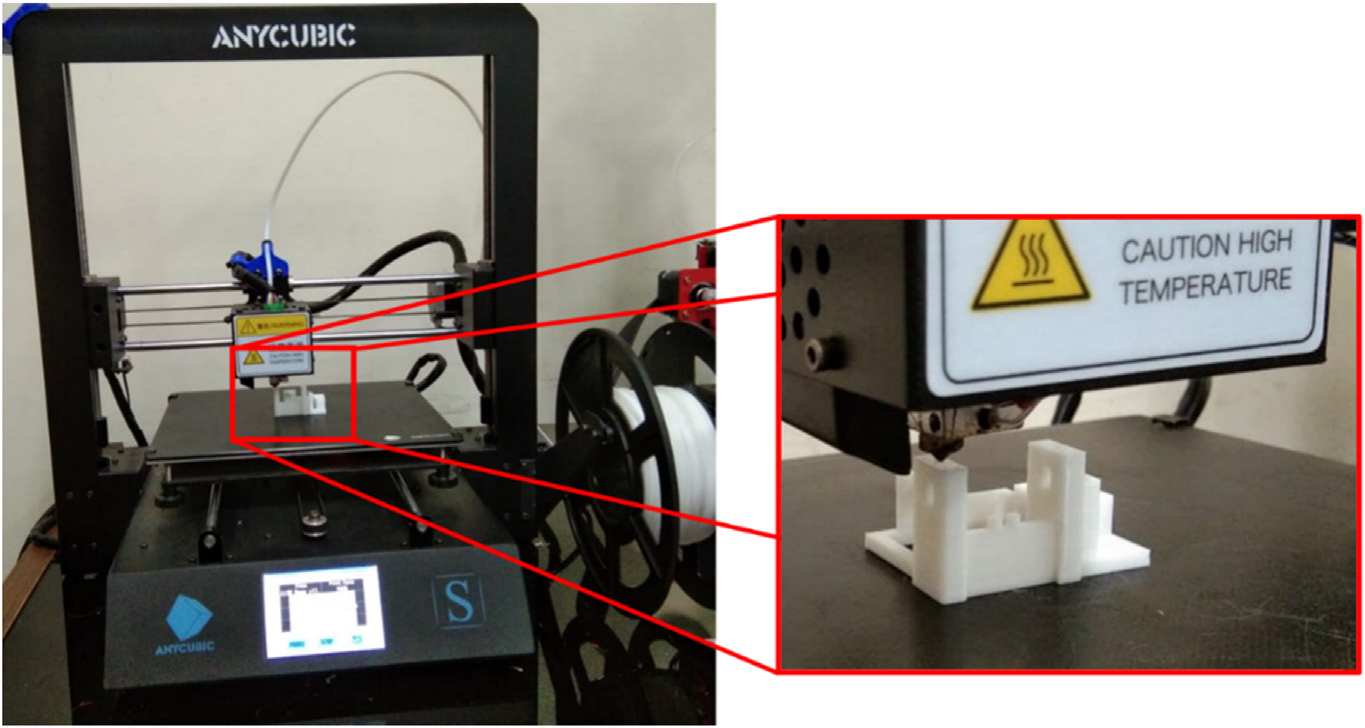
Manufacturing process of the base structure of the music box.

The manufacturing process of the music box's base structure started by identifying the key features and measuring the exact dimension of the music box. These data were then used as a reference for remodeling the music box using CAD software. A 3D printing prototype was made by duplicating the reference music box and assembled with others to ensure accurate and correct measurements. After the accurate measurement was obtained and proven, the remodeling and redesign of the music box were started. The manufacturing process of the base structure is shown in Figure 1. Some features were then added and tested to improve the design. The redesign process aimed to consider the position for each component during assembly and improve the locking mechanism. Determining the coordinate was obtained initially in terms of the further process to change the comb and cylinder. Thus, the model was able to play multiple tunes without any miss-location. The base structure of the music box geometries has a length of 45.3 mm, a width of 36.4 mm, and a depth of 24.4 mm. The printing process of the structures was done on average 75–83 min depends on the model.

**Figure 2:**
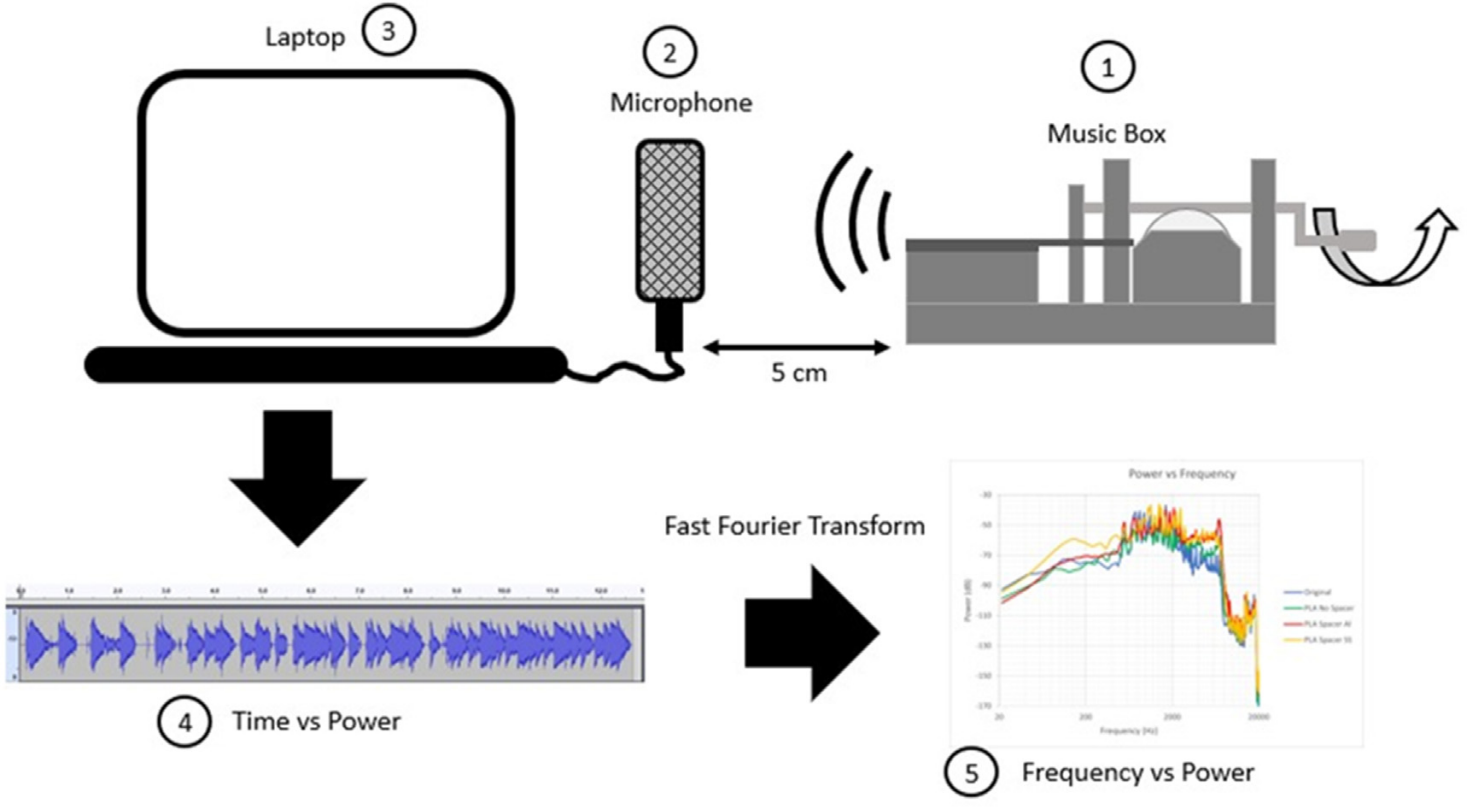
Data acquisition and interpretation process. (1) Music box manually played, (2) Microphone source, (3) Data gathering using computer, (4) Interpretation process, and (5) Data record comparison [27].

After the base structures were printed, the quality of the surfaces was observed using an Olympus U-MSSP4 microscope, and surface roughness was measured using Mitutoyo SJ-210. The surface roughness measurement was done using ISO 1997 standard testing, R Profile measurement, and wavelength (λs) of 2.5 μm. Furthermore, the sound characteristic is then compared with the original music box metal-based structures. The sound characteristic measurement process is shown in Figure 2. The music box was played at the same distance of 5 cm from the microphone. The received sound was then processed in the computer to be compared and analyzed for the different music box models. There are four comparisons in this study which are: original model, PLA model, PLA model with Aluminium spacer, and PLA with Stainless Steel spacer.

## 3 Results and discussion

### 3.1 Surface condition

**Figure 3:**
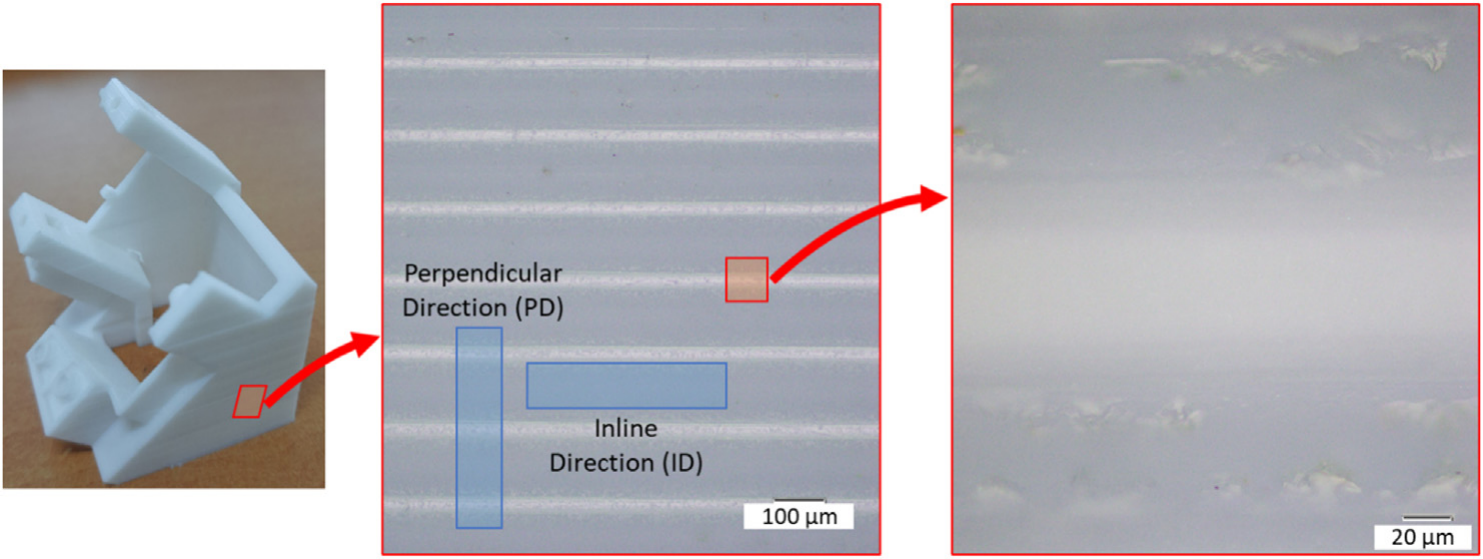
Surfaces condition of the structure after the 3D printing process.

**Figure 4:**
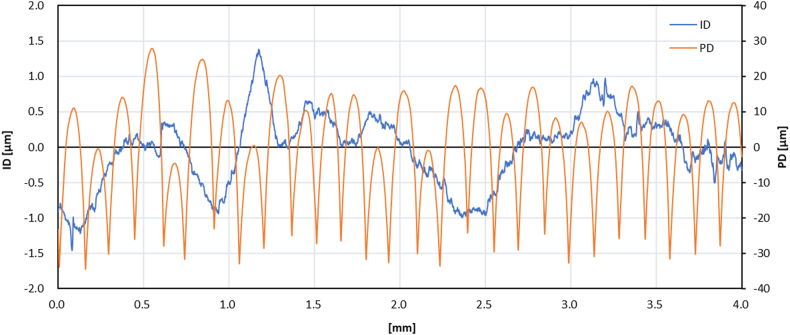
Surface roughness of the sample with inline direction and perpendicular direction from the printing process.

The surface condition of the music box base structure is shown in Figure 3. The printing process used a fused deposition modeling (FDM) method, and the layer-by-layer deposition can be seen in Figure 3. It is shown that each layer has a bonding area that is spotted as white color. The surface roughness of the music box base structure was measured, and the results can be seen in Figure 4. The roughness from perpendicular direction (PD) showed that the layer by layer of deposition could be seen clearly (orange line). In the inline direction (ID), the surface roughness has relatively smoother (blue line). The comprehensive result of surface roughness measurement is shown in Table 4. The surface roughness test was conducted with Lc 0.8 and evaluation length 4 mm. The results show that the Ra value for PD has 20 times higher than ID, with 0.546 μm and 11.230 μm, respectively.

**Table 4:** The surface roughness test result.

Properties	Inline direction	Perpendicular direction
	ID	PD
Ra	0.546 μm	11.230 μm
Rq	0.612 μm	13.477 μm
Rz	1.765 μm	53.955 μm
Rp	1.039 μm	20.883 μm
Rv	0.726 μm	33.072 μm
Rsk	0.066	-0.449
Rkμ	1.809	2.362

### 3.2 Remodeling of the structures

The process of redesign and remodeling of the base structures was not limited to the original design. However, since the original design has several disadvantages, the redesign using the 3D printing method is an opportunity and has advantages in terms of model and speed.

**Figure 5:**
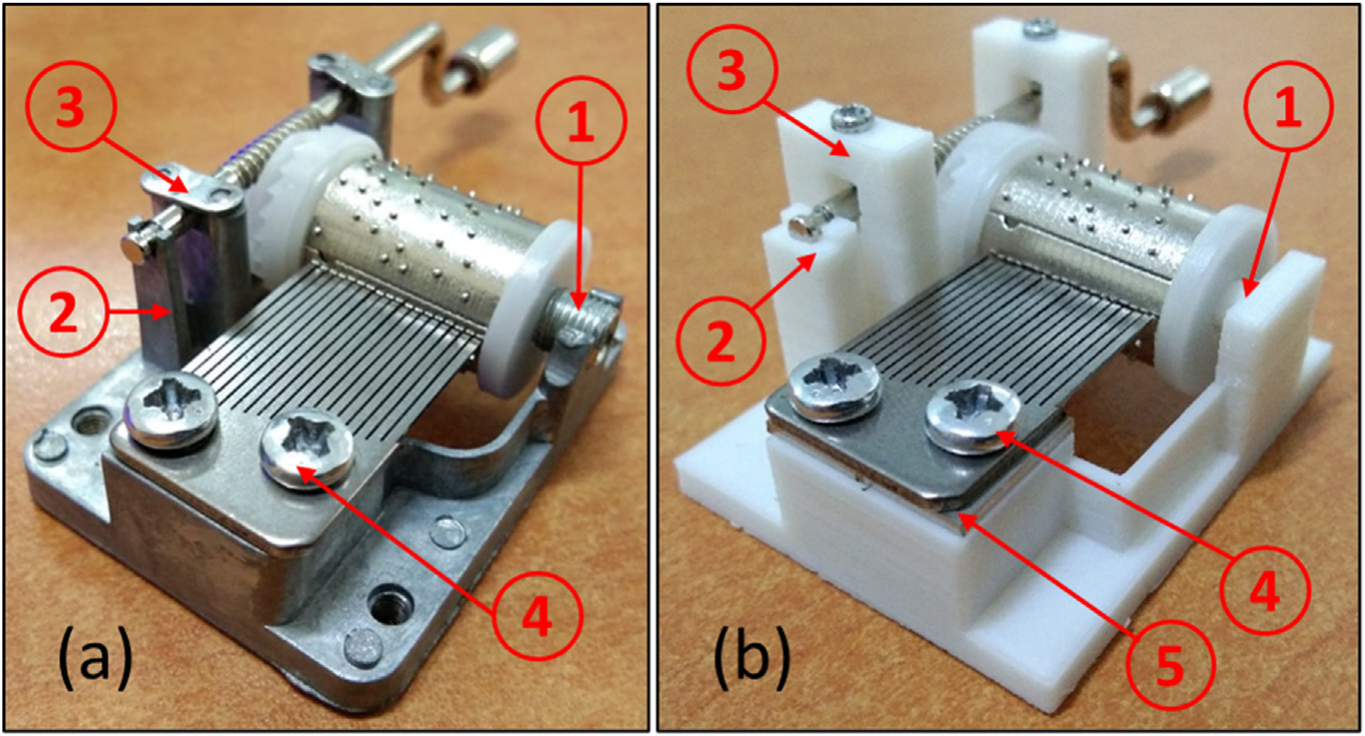
Major modification of the base structure of the music box. (a) Original version (b) 3D Printed version. (1) Barrel mounting, (2) Crankshaft holder, (3) Crankshaft locker, (4) Comb locker, (5) Spacer.

Four different models have been evaluated before choosing the final model. The final model was iterated to eliminate the shortcomings of the original design and also every redesign model. As a result, there are four focus improvements in the present study: barrel mounting, crankshaft holder, crankshaft locker, and comb locker. The pros and cons of each model are listed in Table 5. The components and sections with major modifications are shown in Figure 5, where four main components have been modified, i.e., barrel mounting, crankshaft holder, crankshaft locker, and comb locker.

**Table 5:** Remodelling evolutions.

Model	Barrel Mounting	crankshaft holder	crankshaft locker	comb locker	Advantages	Disadvantages
a	replaced with features printed directly (same dimensions) using 3D Printer	Identical as the original model	Used a 0.5 mm protrusion	Added protrusion	no need for additional bolts in the assembly	Less durable
b	replaced with features printed directly (same dimensions) using 3D Printer	1 mm thickened	Used fit key	Applied with fit key	no need for additional bolts for assembly	Less durable, and the port is easy to break when holding the comb when vibrating
c	replaced with features printed directly (same dimensions) using 3D Printer	coupled with a 1 mm retaining wall	Used a 2 mm bolt	Added a locator with a 3 mm bolt	Have more durable	Used bolts
d	replaced with features printed directly (same dimensions) using 3D Printer	coupled with a 1 mm retaining wall	Used a 2 mm bolt	Add a spacer	Good durability and sound quality	Used Bolts and spacer

**Figure 6:**
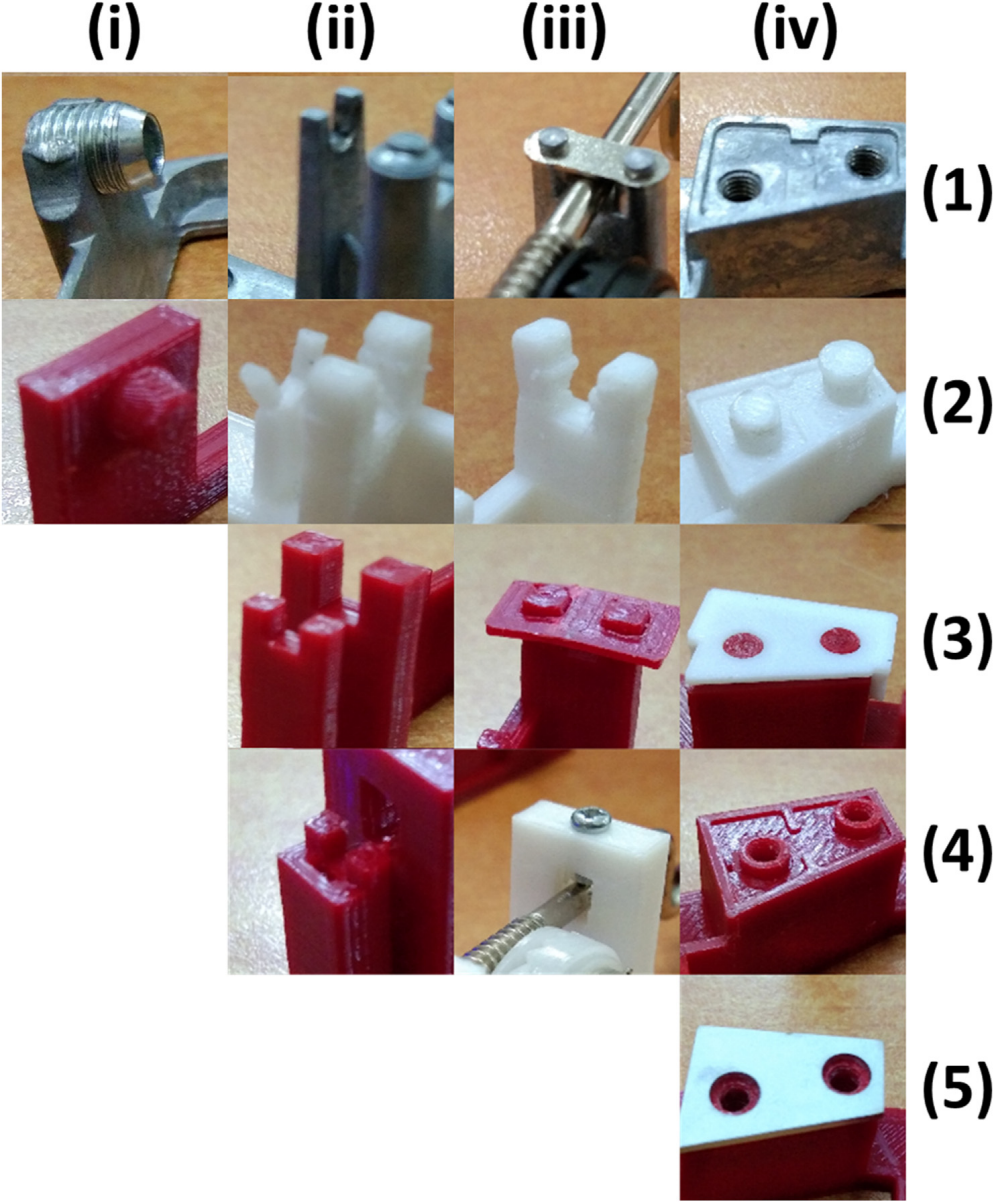
The modification of the music box's base structure based on the function and optimization of each segment. (i) Barrel mounting, (ii) Crankshaft holder, (iii) Crankshaft locker, and (iv) Comb locker.

The music box model evolutions in each segment are shown in Figure 6. It is shown that modification is needed to optimize the model in every part of the base. The first modification is to change the barrel mounting that was previously placed on the cylinder that assembled with a hollow threaded rod (Figure 6(i.1)) by making a feature on the base (Figure 6(i.2)). The results show the music box can be played well, and the structure can hold the components. Even though the lower part of the modified mounting was produced using 3D printing, this part has sufficient strength to support the barrel.

The Crankshaft holder has been modified up to 3 times to ensure the strength of the structure. One of PLA's issues is a lower overall strength than the original models' components made by metal-based material [28, 29, 30, 31], as shown in Figure 6(ii.2). To solve the strength problem, it added specific thicknesses or features on parts that exhibit dynamic loads, such as the wall that prevents lateral movement of the crankshaft at the end of it and the holder itself (Figure 6(ii.3)). The last modification for the music box, as shown in Figure 6(ii.4), was done by enforcing the holder with more strength without putting a lot of weight. The wall is modified by adding the thickness and a cavity to accommodate the crankshaft shape that prevents the barrel from lateral movement.

The original models have two plates that hold the crankshaft permanently, as shown in Figure 6(iii.1). This design prevents changes and disassembles of the model, therefore limiting it to a single tune. The modified model aimed for easy access during the assembly process and tune changes. The first model replaces the plates with a fit lock feature. It is allowed the crankshaft to be mounted with a small amount of force and stays locked while actuated. However, the mounting is snapped after the cyclic assembly process, as shown in Figure 6(iii.2). The PLA plate with two holes was adopted (Figure 6(iii.3)) to prevent the crack of the crankshaft locker. However, the lock system is not stiff enough to hold the crankshaft, and particularly the plated is broken after several mounting-dismounting processes. The modified design based on the bridge structure with bolts inserted was adopted (Figure 6(iii.4)). The locker can perform excellently, and there is no report that the crankshaft was fallen or dislocated.

The comb is attached to the base by two screws on the original model, as shown in Figure 6(iv.1). The problem with this design is that the holes on the comb are 4 mm wide, requiring a delicate hand during the assembly process. After several attempts to reassemble the original model, it is known that the proper contact distance for the pin to plucks the comb's teeth are needed and could vibrate properly. The contact range is between 0.1 mm to 0.25 mm. That means the comb has 0.125 mm to be displaced, either backward or forward, to create pins unable to pluck the comb's teeth and jammed to the cylinder. The first attempt to fix the problem was by creating two 4 mm diameter locators, one for each combs hole, and locked by a bump with a 1 mm wider diameter, as shown in Figure 6(iv.2) comb easily assembled by pushing it down. Unfortunately, this design was failed and cannot be applied because it can break easily once the comb is pushed against it. The following design adopts the same fit key concept as the crankshaft holder (Figure 6(iv.3)). This modification is still inapplicable because the key is unable to hold the comb in place while vibrating. The final design is shown in Figure 6(iv.4), which combines the original model with attempted modification by staying using the same screws to hold the comb and adding two hollow locators to make sure the comb aligns perfectly assembly. The final design is shown in Figure 6(iv.5) to accommodate 1 mm thick metal plates made from aluminium or stainless steel added as a spacer between the comb and the base. This modification aims to improve the sound quality by restricting the comb to touch the base directly and damping its vibration due to PLA properties [32, 33].

**Figure 7:**
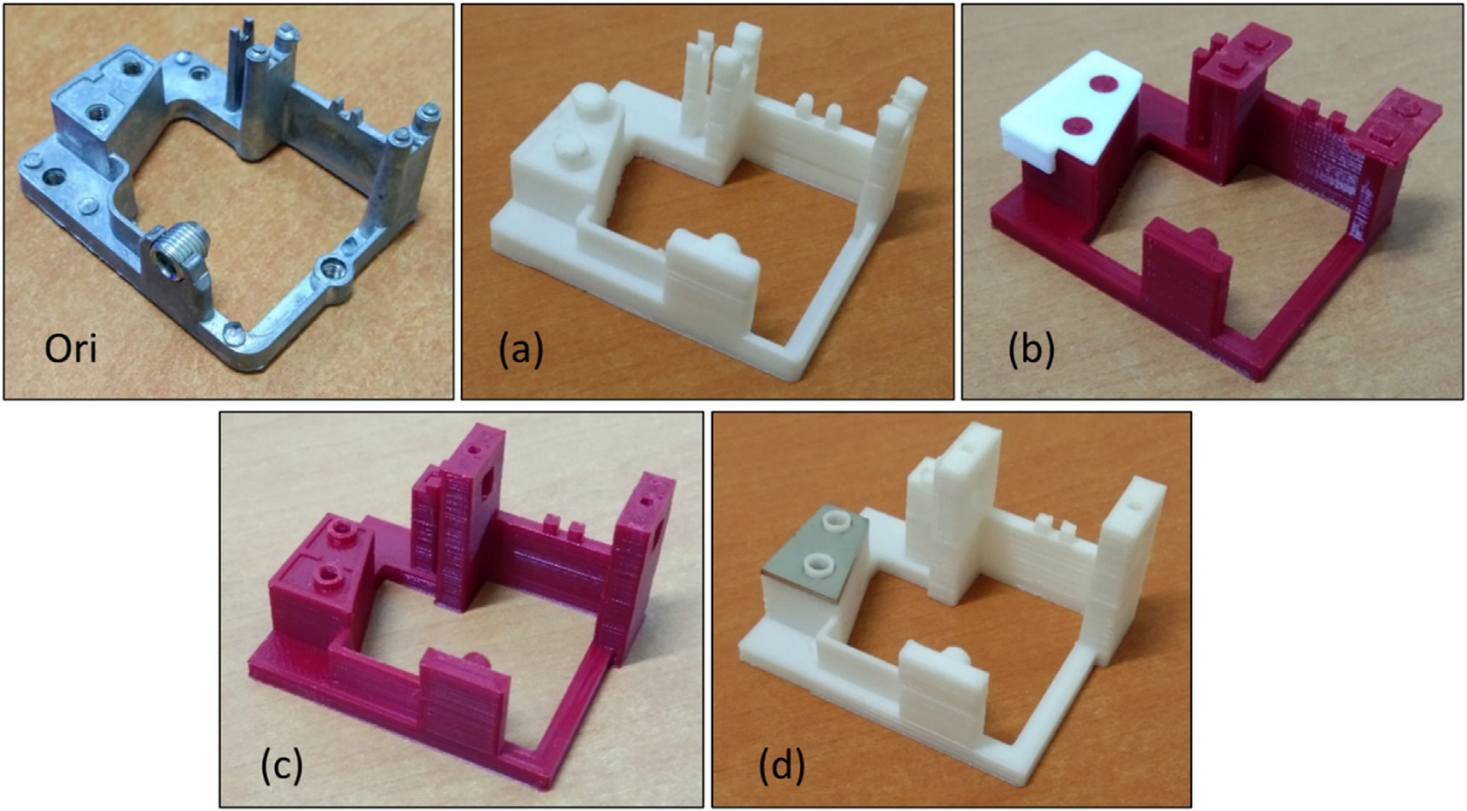
Model evolutions from the original model (metal-based material) upper left and 3D printed models (a–d).

All modifications and evolution of the music box base structures are shown in Figure 7. This process aims to increase the strength of the music box base structures and durability and increase the music box's sound quality. Therefore, one of the essential factors that spacer was inserted on the base structure below the comb is improving sound quality.

### 3.3 Spacer inserter

**Figure 8:**
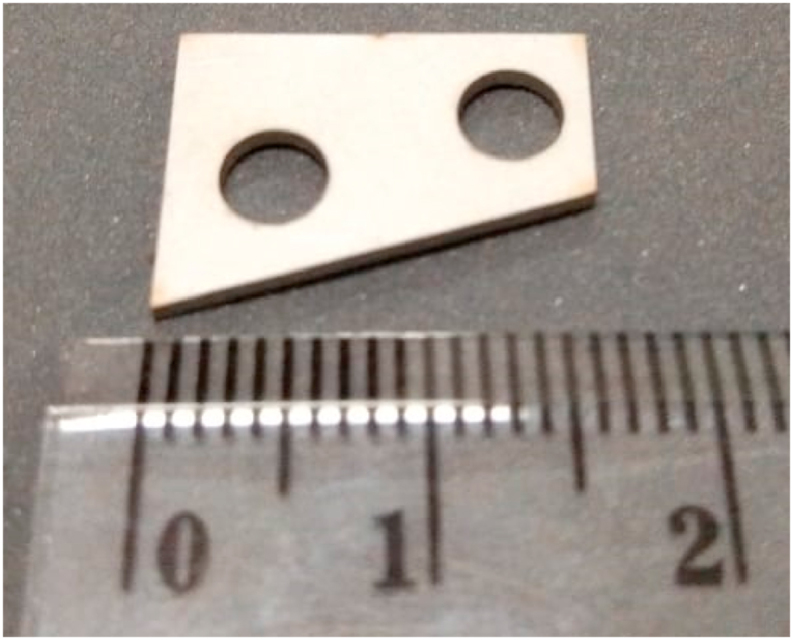
Stainless steel spacer.

Both spacers used in this study have the same geometry and dimension. The geometry resemblance the base structure where the comb is attached. It has the right trapezoid shape, as shown in Figure 8. It also has two 4 mm holes, different from 3 mm holes on the original base model, to accommodate the modified model's added locator feature. These spacers are made from aluminium and stainless steel, but both have the same 1 mm thickness.

Spacers are commonly used to absorb vibration and prevent it from damaging the component [34]. Other than that, as the name suggested, spacers are used to create space between two parts or structures to accommodate the assembling process. But in this study, these spacers were used to preserve the vibration produced by the comb, so the sounds can last longer as it prevents the vibration from being damped by PLA high damping properties.

### 3.4 Manual activation and assembly process

**Figure 9:**
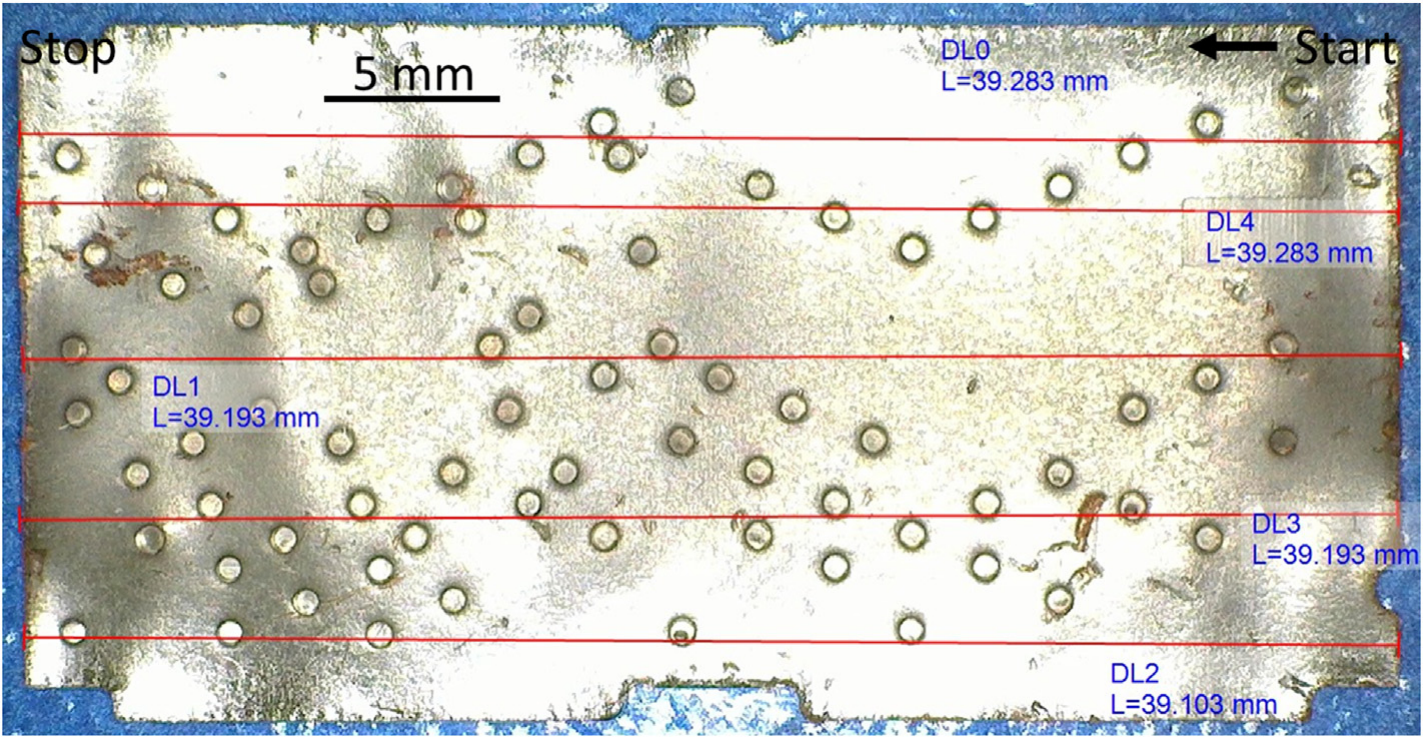
The flatted barrel and the moving direction.

The activation of the music box was used manual process since the music box type is a hand-cranked music box (the playing model used a hand to roll the crankshaft manually). Hence, the activation is started from the part of the barrel where the first point is located. The mapping of the barrel after it flatted can be seen in Figure 9.

All measurement processes started from right to left based on Figure 9. The sound the recorded and analyzed. It is shown that the part of the barrel has a notch located lower right. This mark was used to identify the moving direction of the barrel. The length of the barrel was around 39.2 mm, and the width was 19.7 mm.

The assembly process was also used barehand. The barrel was attached to the based structure model, and then the crankshaft was put in the structure. The comb was then bolted with two bolts before it was manually checked by playing the music box one round. The data recorded when the sound was stable and no dislocation from the barrel.

### 3.5 Sound analysis

**Figure 10:**
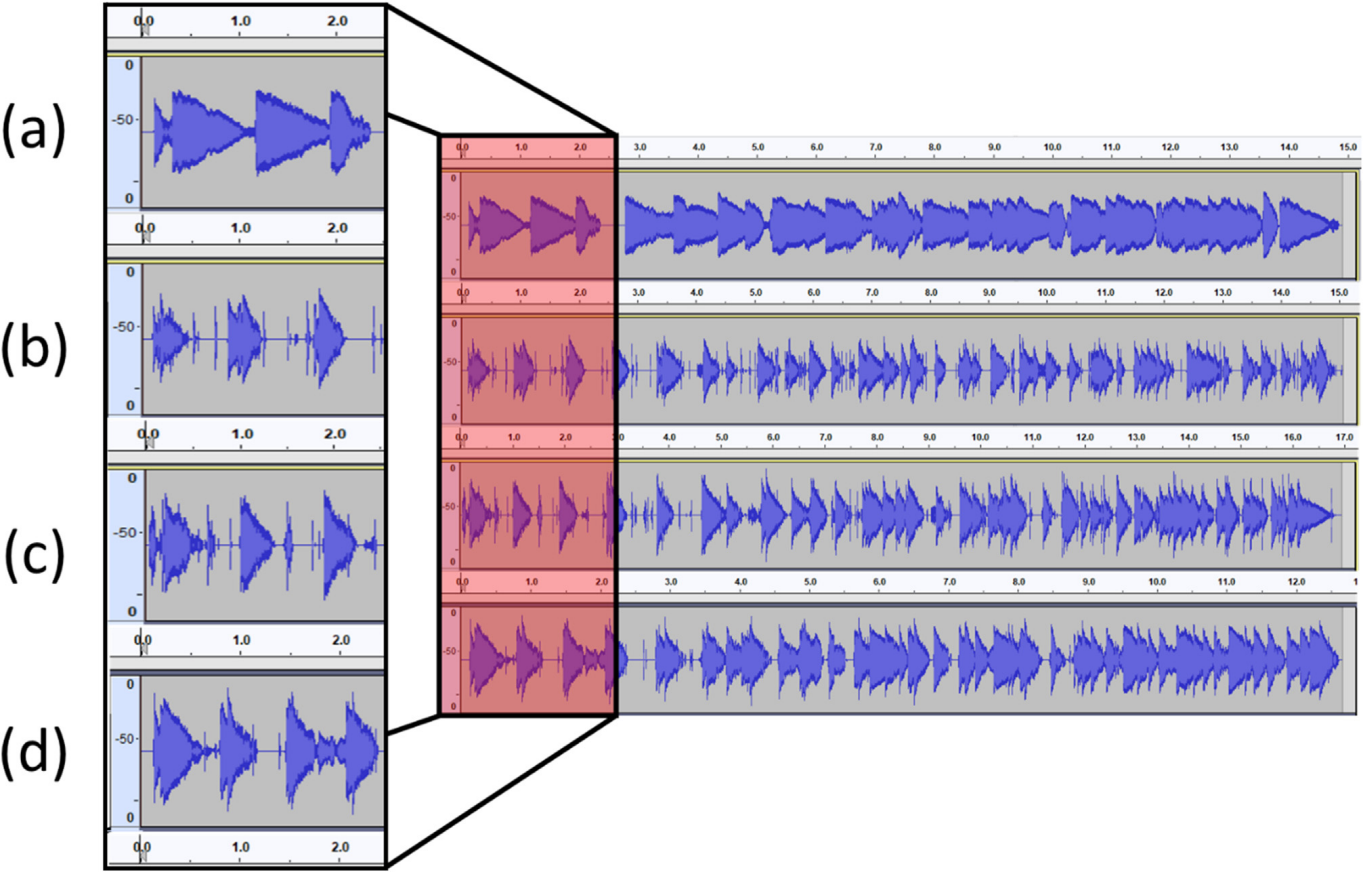
Waveform records, (a) original, (b) PLA no spacer, (c) PLA with aluminium spacer, (d) PLA with stainless steel spacer (The x-axis is time, and the y-axis is power).

The sounds generated by the music box were recorded on a waveform using audacity software. Waveform analysis is a commonly used method to analyzed sound and vibration. This method gives information about when and how loud the sound was, and it can see each tune's peaks and decay time. After the initial data process, the graph can be converted using Fast Fourier Transform to see each frequency dB [35]. The tune played was instead on the higher pitch, so it has high frequencies, and as shown in Figure 10. The x-axis is time, and the y-axis is power (dB). The peaks align show identical and the difference placed in the loudness. The original sound produced with manually played can be downloaded in the Appendix A for original music box base structure from metal-based material, appendix B for PLA-based structure with no spacer, appendix C for PLA-based structure with aluminium spacer, and appendix D for PLA-based structure with stainless steel spacer.

The following are the supplementary data related to this article:

Appendix A The music file (mp3 file) that from original music box base structure from metal-based material. Appendix A

Appendix B The music file (mp3 file) that from PLA-based structure with no spacer (0° angle). Appendix B

Appendix C The music file (mp3 file) that from PLA-based structure with aluminium spacer. Appendix C

Appendix D The music file (mp3 file) that from PLA-based structure with stainless steel spacer. Appendix D

The tune recorded was a glimpse of Canon in D major by Pachelbel. The recorded duration varies from 13 to 17 s. This time variant was due to its music box mechanism that must be actuated by hand. As shown in Figure 10, the model with spacers performs better at most frequencies by producing about seven dB louder sounds than the PLA model without a spacer (Figure 10(b)). It is shown that stainless-steel spacers work slightly better than the aluminium with three dB higher. The results showed that stainless steel and aluminium spacer were able to mimic the sounds produced by the original music box. The point that needs to be considered is the modified model. Although this model produced similar loudness, the sound from PLA no spacer preserved still decays a lot faster than the PLA with spacer, as shown in Figure 10 (b, c, and d).

### 3.6 The angle effect of the music box base structure

**Figure 11:**
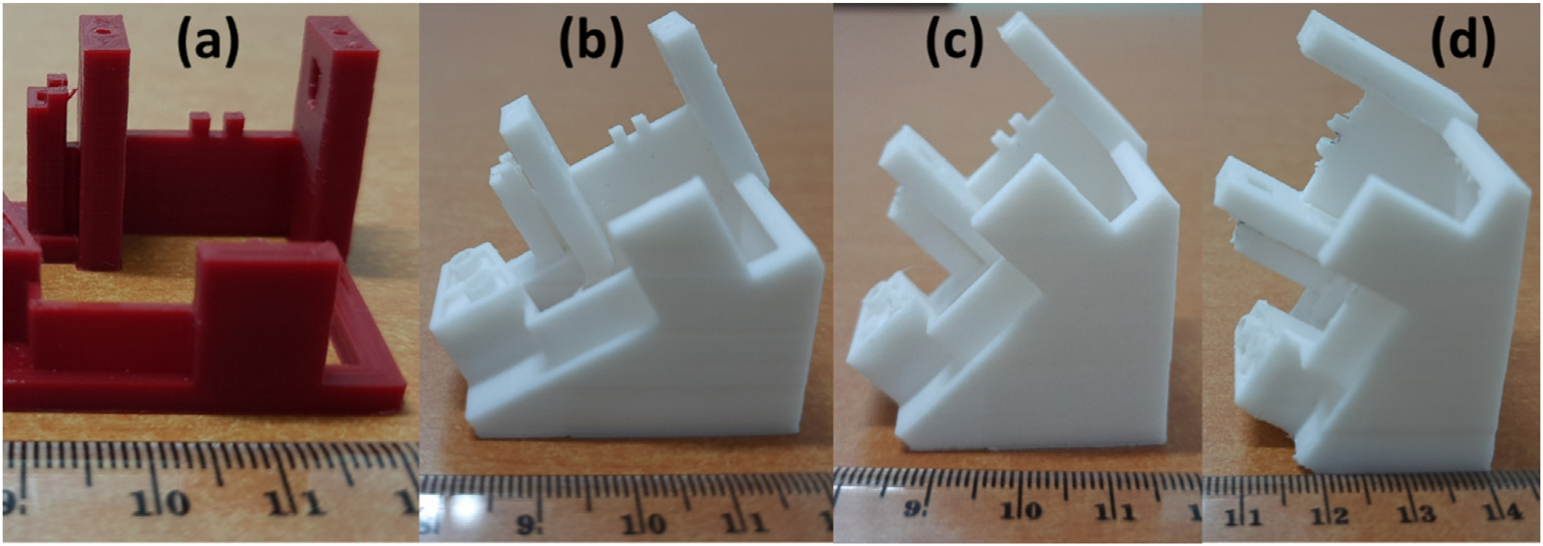
Model of base structures of a music box with a different angle. (a) 0°, (b) 30°, (c) 45°, and (d) 60°.

**Figure 12:**
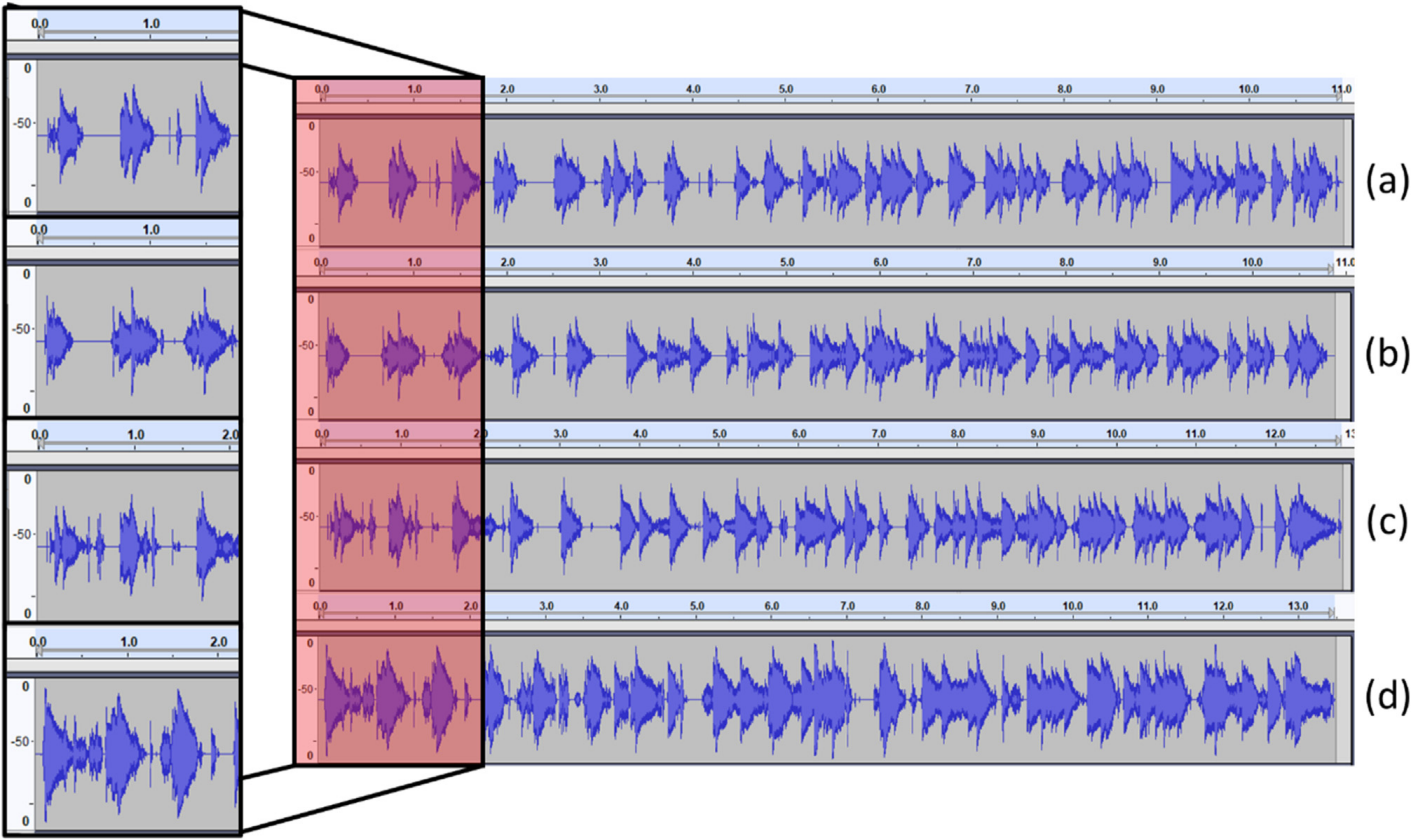
Waveform records. (a) 0°, (b) 30°, (c) 45°, and (d) 60° (the x-axis is time, and the y-axis is power).

The following modification is the music box base structure angle. After the modification was made, the sound quality was examined. The PLA model has added a support structure with a certain angle, as seen in Figure 11. During this modification, the music box base structure has extra walls (area) for the sound to bounce around before escape to the open air [36, 37, 38]. Although the dimensions between those models seen in Figure 11 are different due to the angle effect, the slanted side has the same dimension. Furthermore, this modification was proven to produce a better sound and added sound power level by almost 10 dB on the 60° one, as shown in Figure 12.

These waveforms shown in Figure 12 were acquired by recording the actuated music box five cm from the microphone and processed using Audacity software. The x-axis is time in second and y-axis is power (dB). Some of the sounds were damped due to PLA properties hence shorten the decay time (Figure 12(a–c)). Slanted bases have slower decay due to the extra wall or structure that enables the sounds to bounce and oscillate longer. The 60° slanted model has the longest decay and loudest sound, as shown in Figure 12(d). It is shown that model has a significant difference among the others due to its optimum ratio between the distance from the comb as a vibration source to the wall. Its overall volume matches the natural frequency of the whole system [39, 40, 41]. The original sound produced with manually played can be downloaded in the appendix B for 0° angle, appendix E for 30° angle, appendix F for 45° angle, and appendix G for 60° angle.

The following are the supplementary data related to this article:

Appendix E The music file (mp3 file) that from PLA-based structure 30° angle. Appendix E

Appendix F The music file (mp3 file) that from PLA-based structure 45° angle. Appendix F

Appendix G The music file (mp3 file) that from PLA-based structure 60° angle. Appendix G

### 3.7 Discussion

The discussion in the present paper has two parts, sound quality comparison, and weight and cost comparison.

#### 3.7.1 Sound comparison

The modification of the music box model dimensions was made to assist the printer and filament properties, which affected the expansion of the overall dimensions from 0.1 to 0.3 mm and holes shrinkage that led to up to 0.5 mm difference. Therefore, after the redesign process was finished, four models were compared: the original model, the PLA model, and two PLA models with a 1 mm spacer (aluminium and stainless steel).

**Figure 13:**
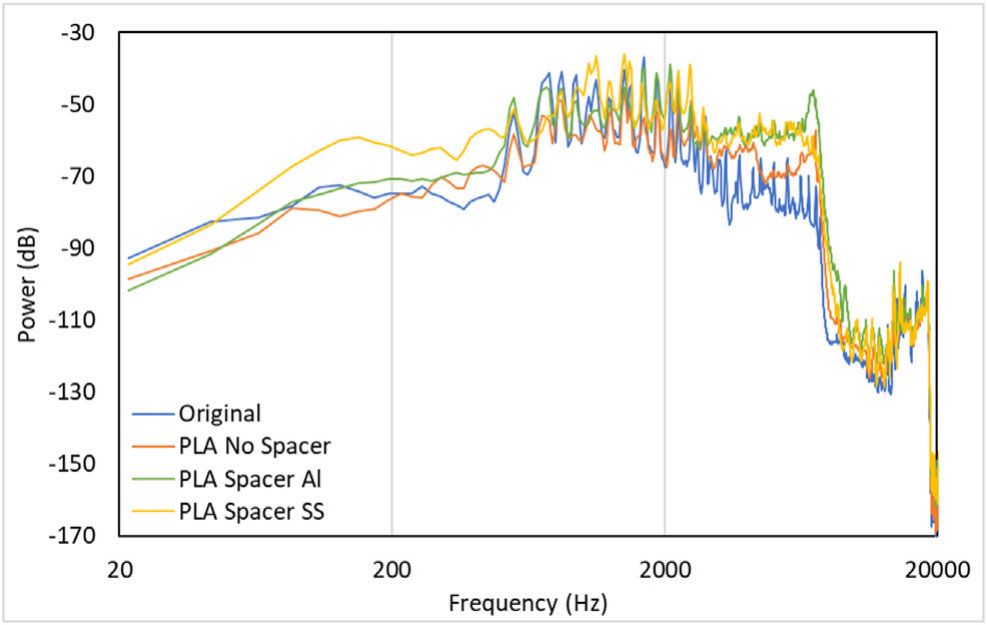
Recorded sounds spectrum data acquisition with a different model of additional spacer.

**Figure 14:**
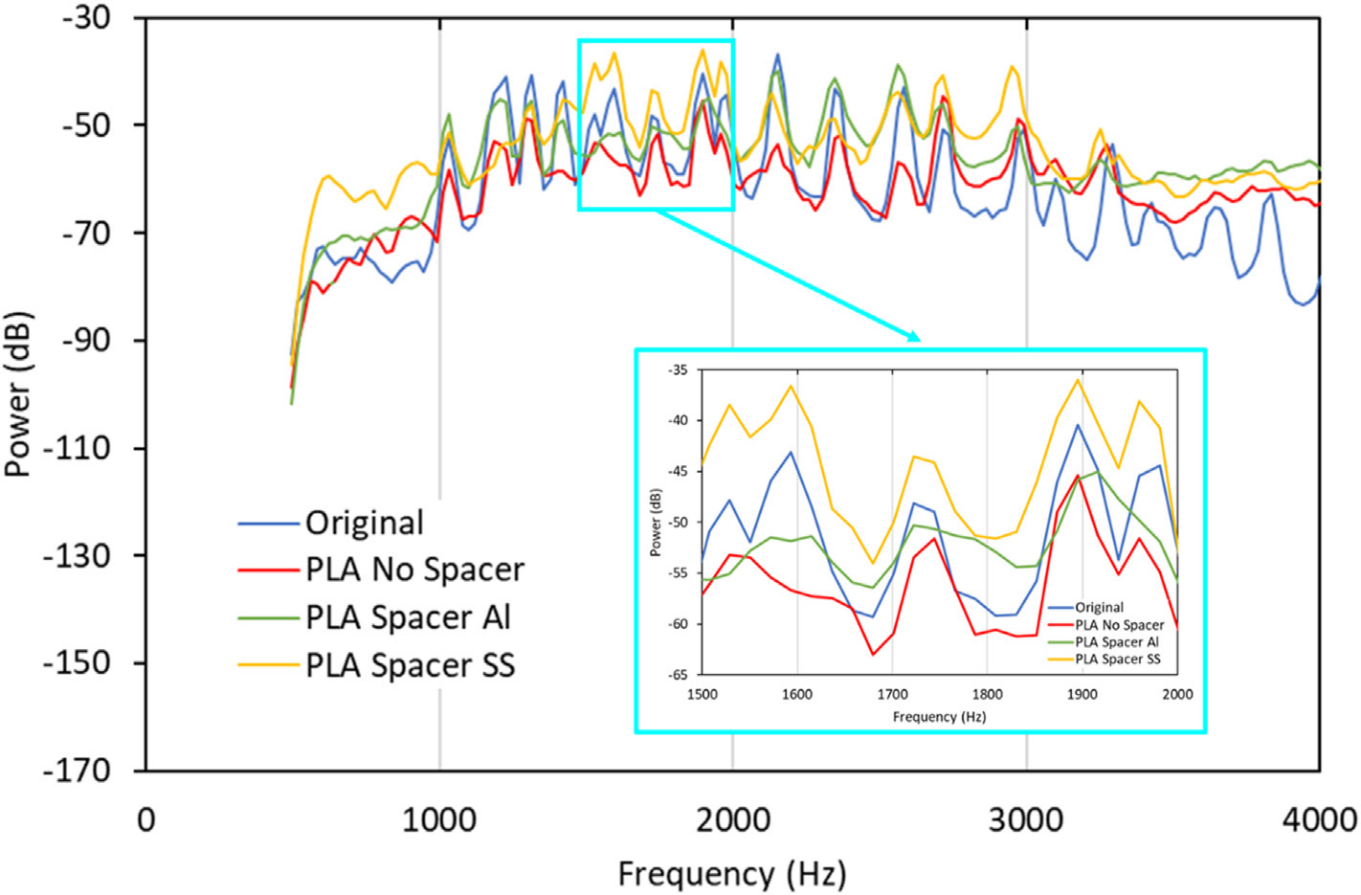
Recorded sounds spectrum at human most sensitive frequencies (500–4000) with a different additional spacer model.

The results have shown that the peak of the sound was slightly different on some occasions since it was actuated by hand. The all-PLA model's sound damped the fastest among all models. This occurred due to PLA high damping. Figure 13 illustrates the power (dB) vs. Frequency (Hz) from all record data. The frequency range from 500 Hz to 4000 Hz was presented in Figure 14. The present frequency was used in the present study because of the range where human hearing is most sensitive. However, at higher and lower frequencies, the power level has distinct differences [42].

It is shown that the comparison between the original type and the PLA-based types have higher decibel compared to the original. The higher the decibel level, the louder the noise. Moreover, Petitot et al. [46] investigated the influence of modulation and carrier frequency on steady-state auditory responses (ASSR). The ASSR identification limits overestimate behavioral thresholds for traditional audiometric (carrier) frequencies between 500 and 4000 Hz. Moreover, Hongisto et al. [41] did the experiment related to hearing ability of the human. They used the Hughson-Westlake method in frequencies 250, 500, 1000, 2000, and 4000 Hz in both ears using Micromate 304, Madsen Electronics Ltd., USA. In the frequency range between 250 and 4000 Hz, the normal hearing was characterized as pure-tone thresholds exceeding the average hearing threshold curve by less than 20 dB and related to the output frequency obtained from the various types of a music box. Figure 14 shows that the stainless spacer PLA-based structures tend to have higher sound quantities than the original model in the frequency range between 1500 to 2000. These results occurred due to the damping value of metal smaller than only PLA-based structures, with the highest being an aluminium spacer. The results agree with Orban [43], and Meveda and Patel [44], where they investigated the damping properties of different materials. The results show that polymer-based materials have a damping ratio of 0.01–0.5 [25, 43] and steel-based material with 0.0069, and aluminium with 0.0035 [44]. This is clear that the sound quality improved in the spacer model due to the damping material of metal-based structures.

**Figure 15:**
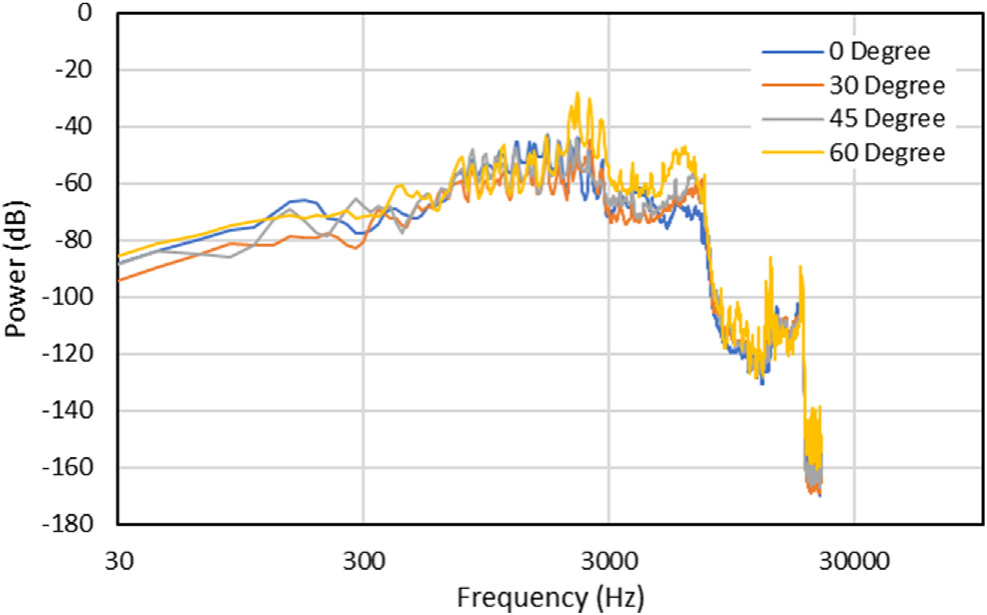
Recorded sounds spectrum data acquisition with different angles.

**Figure 16:**
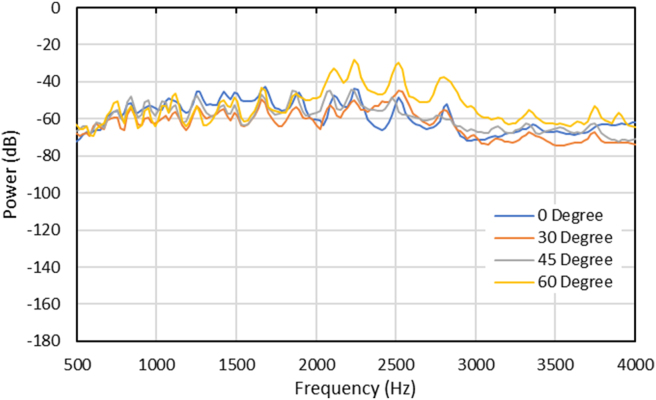
Recorded sounds spectrum at human most sensitive frequencies (500–4000 Hz) with different angles.

Regarding the effect of the music box base structure angle on the sound quality, the 60° base structure angle is relatively higher than the other angle, as shown in Figure 15. In terms of sound analysis, frequency analysis was commonly used as it is possible to see each effect on the observed object. In Figure 15, it can be identified that the 0° model music box frequency and the modified are aligned almost identically. This is because the music box plays the same tune. Furthermore, the power of all degrees was also similar, although it differs on some frequencies. The detailed power vs. frequency graph in the specific frequency (500–4000 Hz) can be seen in Figure 16.

**Figure 17:**
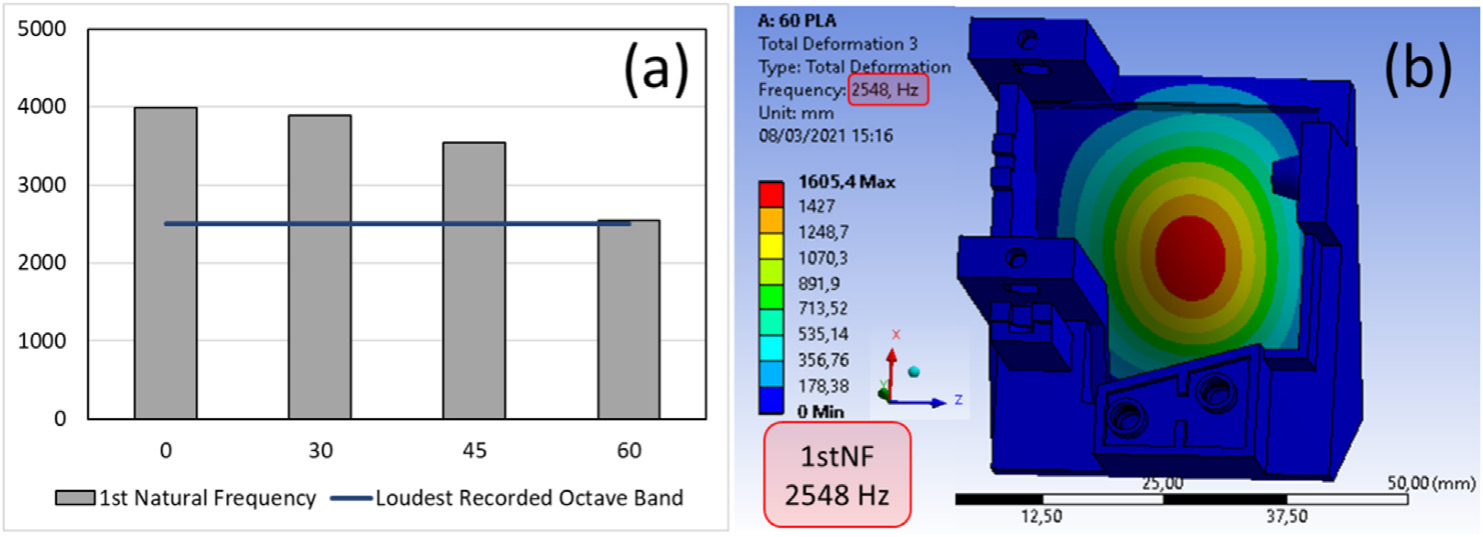
(a) 1^st^ Natural frequency comparison between angle effect, (b) loudness recorded of octave band from simulation.

After further study through the modal simulation in a specific frequency (Figure 16), it is found that the 60° base structure angle produced the loudest recorded sound at the 2500 octave range. This condition was then analyzed by using simulation analysis to confirm the findings. From the simulation shown in Figure 17, the first natural frequency occurred at 2548 Hz, with the deformation primarily located at the wall structure. It is to be noted that the displays of the magnitude from displacement have no meaning and do not represent the actual specimen's displacement value. This condition is because there is no applied load (0 N) to the structure in the modal analysis.

Moreover, the results were compared between the experiment using Ansys FEM software and modal analysis to determine the 1st natural frequency of each angle. The results in Figure 17 is shown that the 60° base structure angle has the closest result with the experiment. This is why the 60° base structure model can produce the loudest recorded sound because this model has a natural frequency that is identical to the music box after playing.

The present research shows that the 3D printing method can be used to manufacture music instruments with excellent results in terms of speed and accuracy. The results were also exposed that the present study can be used for musicians, researchers, and engineers related to music instruments deals with 3D printing.

### 3.8 Weight and cost comparison

**Figure 18:**
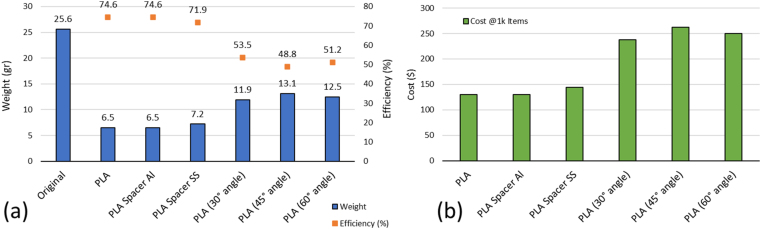
Weight and cost efficiency comparison of the different music boxes from PLA-based structures.

After the model printed using 3D Printer, the weight of the base structures then measured using weight balance. The weight comparison between original and the modified models were showed in the Figure 18(a). For the comparison, comb, barrel, crankshaft, and locker were excluded from the measurement process. It is shown that the weight reduction can be achieved. The spacer was included in the weight measurement. Thus, the results show that by using SS spacer, the weight was higher compared with Al spacer. It is shown that the efficiency can be reach with the most efficient reach 74.6% and the less efficient was 48.8% from PLA original, and PLA (45° angle), respectively. The comparison of the model based on the cost of the source material (PLA) that can be safe during the manufacturing process can be seen in Figure 18(b). The data based on the assumption that 1 kg cost of PLA filament is $20. It is shown that PLA with spacer have higher efficiency in the cost compared with the other model, while the highest cost is occurred for base structure with 45° angle. The weight and cost comparison has shown that the PLA model can be set tobe more efficient and create cost efficient.

### 3.9 Sound quality analysis

The sound quality has been used by many researchers to evaluate different applications i.e., Automotives [45, 46, 47], Diesel engines [48], and power plants [49]. Lim [45] investigated that the sound quality can be evaluated quantitatively using the frequency vs. time graph, where the sound was measured based on the peak loudness that occurred during measurement. A different method was proposed by Nykänen and Sirkka [46] that used power (dB) and frequency to evaluate the quality of automobile power windows. In their study, sound quality was classified into several categories based on subjectivity. For instance, the power window sound was categorized by steady, load, and dull. Power vs. frequency was used by Sellerbeck et al. [48] to evaluate the sound quality from diesel engines. The study also analyzed the sound quality based on the modulation spectrum that was measured during the test. A different peak from the spectrum (Power vs. Frequency) was established. A similar evaluation was used by Aoki et al. [49] in the power plant case.

Based on the previous study, the different evaluation was used to evaluate the sound quality of the mechanical systems in a different application. Hence, the present study used power vs. time and power vs. frequency to evaluated the sound quality by checking its peak and the waveform record. The decay time was also checked. Faster decay time showed that the sound of the music box is not resonated last longer. The present study showed that the effect of the spacer made from metal could significantly improve the decay time and peak loudness, as shown in Figures 10 and 14.

## 4 Conclusion

**Figure 19:**
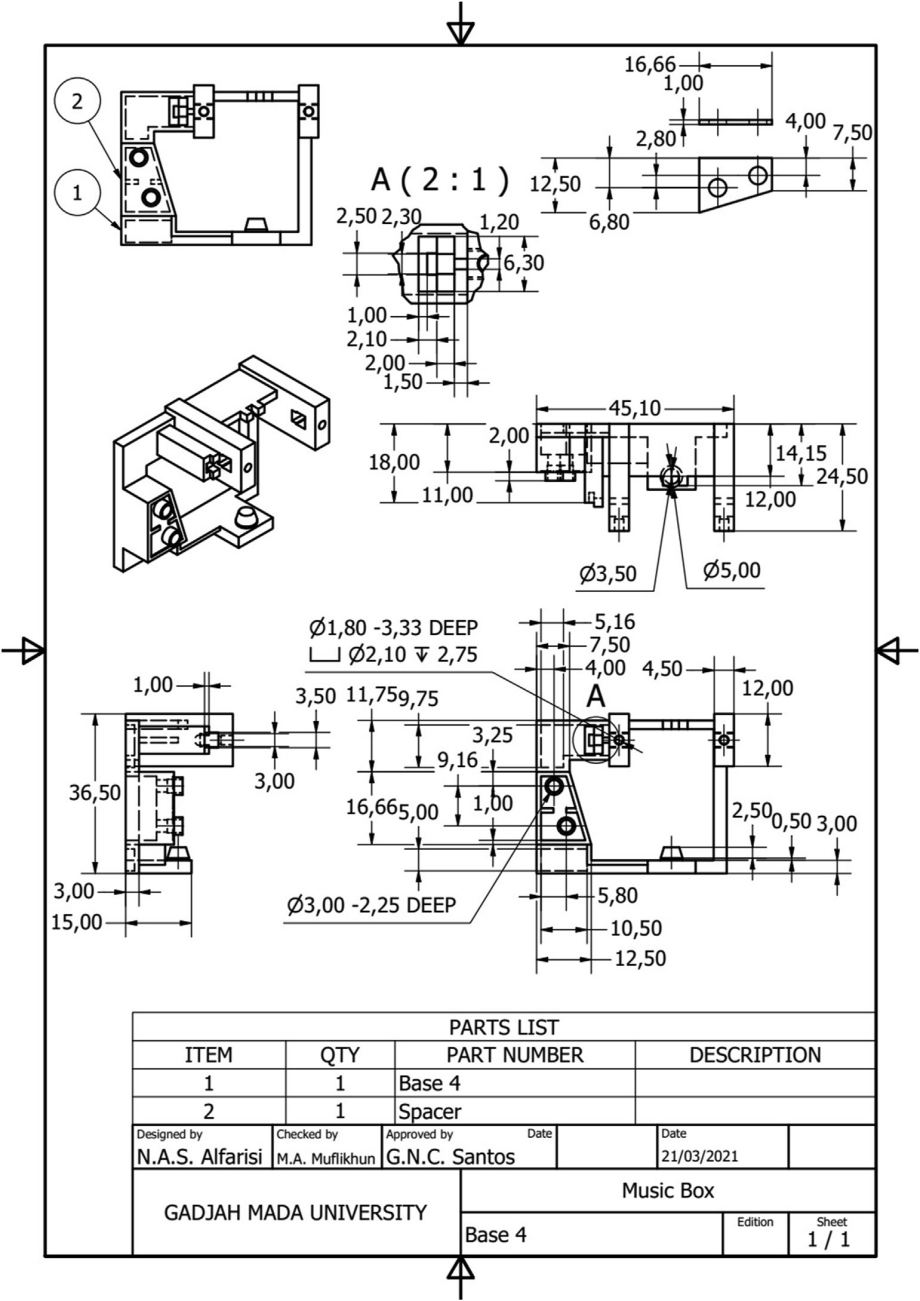
Detail drawing of music box based structure.

Producing musical instruments with 3D printing have a wide range of possibilities. Its ability to create complex shapes with high accuracy and precision is essential in the manufacturing process of musical instruments. Moreover, the improvement of the model can be easily applied where the material source has an affordable price compared with the metal manufacturing process. The present study successfully remodeling and redesigned the base structure of a hand-cranked music box using 3D printing. The detailed based structure of music box can be seen in Figure 19. The study showed four models were achieved, and four improvements were needed in the Barrel mounting, Crankshaft holder, Crankshaft locker, and Comb locker. Moreover, it is shown that using 3D printing and the manufacturing process can be built more accessible where the pinned cylinder and comb can be changed more straightforwardly. The results show that the model from 3D printing was proven and has a similar sound spectrum with the original at frequencies where human hearing is most sensitive (500–4000 Hz). The current study showed that the sound quality could be improved by using PLA and added metal spacer, and PLA's high damping ability can be fixed. Moreover, the angle effect of the sound quality was also evaluated where the 60° slope exhibited the loudest and best sound quality.

## Declarations

### Author contribution statement

Muhammad Akhsin Muflikhun: Conceived and designed the experiments; Performed the experiments; Analyzed and interpreted the data; Contributed reagents, materials, analysis tools or data; Wrote the paper.

Naufal Achmad Salman Alfarisi: Performed the experiments; Analyzed and interpreted the data; Wrote the paper.

Rachmadi Norcahyo, Jayan Sentanuhady, Nikmatul Azizah & Gil Nonato C. Santos: Analyzed and interpreted the data; Wrote the paper.

### Funding statement

This work was supported by the Department of Mechanical and Industrial Engineering, 10.13039/501100012521Gadjah Mada University (NASA, RN, JS, MAM), and 10.13039/100012938De La Salle University, Manila (GNCS).

### Data availability statement

Data will be made available on request.

### Declaration of interests statement

The authors declare no conflict of interest.

### Additional information

Supplementary content related to this article has been published online at https://doi.org/10.1016/j.heliyon.2021.e08432.
